# San-Huang-Xie-Xin-Tang Constituents Exert Drug-Drug Interaction of Mutual Reinforcement at Both Pharmacodynamics and Pharmacokinetic Level: A Review

**DOI:** 10.3389/fphar.2016.00448

**Published:** 2016-11-28

**Authors:** Jiasi Wu, Yingfan Hu, Li Xiang, Sheng Li, Yi Yuan, Xiaomei Chen, Yan Zhang, Wenge Huang, Xianli Meng, Ping Wang

**Affiliations:** ^1^Chengdu University of Traditional Chinese MedicineChengdu, China; ^2^Chengdu Institute of Biology, Chinese Academy of SciencesChengdu, China; ^3^Hainan Medical UniversityHainan, China

**Keywords:** San-Huang-Xie-Xin-Tang, constituents, anti-inflammatory, NF-κB, MAPK, JAK/STAT, intestinal transporter

## Abstract

Inflammatory disorders underlie varieties of human diseases. San-Huang-Xie-xin-Tang (SHXXT), composed with *Rhizoma Rhei* (*Rheum palmatum* L.), *Rhizoma Coptidis* (*Coptis chinensis* Franch), and *Radix Scutellaria* (*Scutellaria baicalensis* Georgi), is a famous formula which has been widely used in the fight against inflammatory abnormalities. Mutual reinforcement is one of the basic theories of traditional Chinese medicine. Here this article reviewed and analyzed the recent research on (1) How the main constituents of SHXXT impact on inflammation-associated signaling pathway molecules. (2) The interaction between the main constituents and efflux pumps or intestinal transporters. The goal of this work was to, (1) Provide evidence to support the theory of mutual reinforcement. (2) Clarify the key targets of SHXXT and suggest which targets need further investigation. (3) Give advice for the clinical use of SHXXT to elevated the absorption of main constituents and eventually promote oral bioavailability. We search literatures in scientific databases with key words of “each main SHXXT constituent,” in combination with “each main inflammatory pathway target molecule” or each main intestinal transporter, respectively. We report the effect of five main constituents on target molecules which lies in three main inflammatory signaling pathways, we as well investigate the interaction between constituents and intestinal transporter. We conclude, (1) The synergistic effect of constituents at both levels confirm the mutual reinforcement theory of TCM as it is proven in this work. (2) The effect of main constituents on downstream targets in nuclear need more further investigation. (3) Drug elevating the absorption of rhein, berberine and baicalein can be employed to promote oral bioavailability of SHXXT.

## Introduction

Inflammation, a complex response triggered by pernicious stimuli like pathogens or irritants, verified to be involved in process of many diseases such as Alzheimer Disease, type 2 diabetes, rheumatoid arthritis, etc., (Chiapinotto Spiazzi et al., 2015; Garimella et al., 2015; Saito et al., 2015). Generally, inflammation is classified as acute and chronic type. Acute type only last a few days with neutrophil infiltration, while chronic type can last up to years with infiltrations of lymphocytes and macrophages (Ambrozova et al., 2016). Inflammatory pathways perform a crucial part for signal transduction and recent research provide genuine evidence showing NF-κB, MAPK and JAK/STAT are the three main pathways (Bertolini, 2012; Ottani et al., 2015).

As a famous traditional Chinese medicine (TCM) formula which has been used for centuries, San-Huang-Xie-Xin-Tang (SHXXT) displays good curative activation in the treatment of inflammatory disorders such as atherosclerosis (Wang Y. S. et al., 2011), upper respiratory tract infection (Ma et al., 2009; Kim et al., 2014), diabetic nephropathy (Wu et al., 2015), gastritis, gastric bleeding and peptic ulcers (Lo et al., 2005), and these protective effects are correlated with reactions of weakening inflammatory by suppressing cytokine/chemokine production. SHXXT has a quite simple composition with only three herbals, namely *Radix et Rhizoma Rhei* (*Rheum palmatum* L.) [RR, yields anthraquinones like emodin(Emo), rhein(Rhe) and aloe-emodin (Aem)], *Rhizoma Coptidis* (*Coptis chinensis* Franch) [RC, yields alkaloids like berberine(Ber) and coptisine(COP)], *and Radix Scutellaria* (*Scutellaria baicalensis* Georgi) [RS, yields flavonoids like baicalin(Bai) and baicalein (Bae)]. Previous studies show the basic effective constituents of SHXXT responsible for the anti-inflammatory effect may be Ber, Bai, Emo, Rhe, and Aem (Ma et al., 2009), plus, Bae is considered as a quality control indicator of RS (Zhang et al., 2013b). In regard of the bioavailability of SHXXT, A rapid and sensitive UPLC-ESI/MS method determined 17 active SHXXT constituents with good linearity in a relatively wide concentration ranges, among which, Bai is the most abundant. In bloodstream, the major forms of SHXXT include Bae, Emo, Aem and Rhe, while only the parent form of Rhe can be detected, and the conjugated effect may be accounted for their physicochemical property differences (Li et al., 2010; Shia et al., 2011).

Intestinal transporters (IT), such as P-gp, MRP, BCRP (Sampson et al., 2015), SGLT1 (Asano et al., 2004) and OCT (Bader et al., 2014), play a critical role in the process of intracellular and efflux transport. Numerous evidence illustrate the main constituents in SHXXT are the substrates of efflux transporters which leads to a very low oral bioavailability (Huang S. et al., 2011; He et al., 2014; Wei et al., 2014; Di et al., 2015). However, most studies only concentrate on solitary constituent, whether they have mutual effect on respective absorption remains to be elucidated.

There's growing evidence indicating that all those constituents above, while exclusively dosed, possess anti-inflammation effect by affecting a variety of target molecules in signaling pathways (Shih et al., 2007; Hamsa and Kuttan, 2012; Zhang et al., 2013a; Hu et al., 2014). We are all clear that, Chinese herbal combination should not only improve curative effects and reduce side effects, but also promote the mutual absorption of effective constituents. In this study, we review the recent studies and discuss how the three classic herbals of SHXXT, RS, RR, and RC, reach the goal of synergistic interaction at both pharmacodynamics and pharmacokinetic level.

## Pharmacodynamic level

### Effect of the active constituents on molecules in NF-κB pathway

TLR-4 is the first described TLRs in mammals, it responds to LPS which can trigger NF-κB activation and pro-inflammatory cytokines secretion (Lee et al., 2010), constituents that can block the binding between TLR-4 and LPS are supposed to be valued in inflammation treatment (Wu et al., 2016). As summarized in Table 1, It is reported that Ber, Bai and Rhe exert inhibitory effect on TLR-4 expression in varies of models (Lee et al., 2010; Li et al., 2011; Cabrera-Benitez et al., 2012; Hou et al., 2012; Chen C. C. et al., 2014; Chen et al., 2015), and the combination of TLR-4 and LPS is observed to be blocked by Ber (Jeong et al., 2014). So, it seems that the anti-inflammatory mechanism of SHXXT begins at a really early stage, ever since LPS are interacting with upstream membrane protein.

**Table 1:** **Effect of the active constituents on molecules in NF-κB pathway**.

**Target**	**Animal or cell culture**	**Model building**	**Control (P or N)**	**Drug**	**Dose**	**Treat time**	**Result**
TLR-4	Microglial cells	IL-1β	Vehicle	Ber	50 μM	24 h	TLR-4 expression↓ Chen C. C. et al., 2014
	BALB/c mice	LPS	Yohimbine	Ber	50 mg/kg	3 d	TLR-4 mRNA expression in ileum tissue↓ Li et al., 2011
	C3H/HeN,C3H/HeJ mice	TNBS	Vehicle	Ber	10–20 mg/kg	3 d	TLR-4 expression in colonic epithelial cell↓ Lee et al., 2010
	PM cell	LPS	Mangiferin	Ber	10,20 μM	1 h	TLR-4 & LPS banding↓ Jeong et al., 2014
	Microglial cells	OGD	Vehicle	Bai	40,20.10 ug/ml	24 h	TLR-4 mRNA expression↓ Hou et al., 2012
	IgAN SD rats	BSA, LPS, and CCl_4_	Vehicle	Rhe	400 mg/kg/d	6 w	TLR-4 expression in renal↓ Chen et al., 2015
	BEAS-2B cell	LPS	CKT0103	Rhe	10 μM	18 h	TLR-4 level↓ Cabrera-Benitez et al., 2012
	BALB/c mice	LPS	TAK-242	Rhe	100 mg/kg		TLR-4 expression↓ Zhang et al., 2015
	Wistar rats	LPS	Vehicle	Emo	10mg/kg/hr	1,2 h	TLR-4 expression↓ Li A. et al., 2013
MyD88	Microglial cells	IL-1β	Vehicle	Ber	50 μM	24 h	MyD88 expression↓ Chen C. C. et al., 2014
	Microglial cells	OGD	Vehicle	Bai	20 ug/ml	2 h	MyD88 activation↓ Hou et al., 2012
	C57BL/6 mice	DSS	Mesalazine	Bai	100 mg/kg/12 h	7 d	colon MyD88 expression↓ Feng et al., 2014
	ICR mice	Placebo	Ribavirin	Bai	375 mg/kg/d	7 d	MyD88 mRNA expression↓ Wan et al., 2014
TNFR1	HEK293 cell	TNF-α	None	Ber	25 μmol/L	24 h	TNFR1gene expression↓ Pandey et al., 2008
TRADD	HEK293 cell	TNF-α	None	Ber	25 μmol/L	24 h	TRADD gene expression↓ Pandey et al., 2008
TRAF2	HEK293 cell	TNF-α	None	Ber	25 μmol/L	24 h	TRAF2 gene expression↓ Pandey et al., 2008
	SAP SD rats	ST	SO	Emo	30 mg/kg	6 h	TRAF2 protein expression↓ Wu et al., 2013
TRAF6	Microglial cells	OGD	Vehicle	Bai	40 ug/ml	4 h	TRAF6 protein level↓ Hou et al., 2012
NIK	HEK293 cell	TNF-α	None	Ber	25 μmol/L	24 h	NIK gene expression↓ Pandey et al., 2008
	Fischer 344 rats	“Age” diet	Young rats	Bai	10,20 mg/kg/d	10 d	NIK phosphorylation↓ Kim et al., 2006
Raf	Fischer 344 rats	“Age” diet	Young rats	Bai	10,20 mg/kg/d	10d	Raf phosphorylation↓ Kim et al., 2006
	U251/U87 cell	None	Vehicle	Ber	15 μM	1-7 d	p-Raf phosphorylation↓ Liu et al., 2015
IRAK1	PM cell	LPS	Mangiferin	Ber	10,20 μM	90 min	phosphorylation of IRAK1↓ Jeong et al., 2014
IKK	PM cell	LPS	Mangiferin	Ber	10,20 μM	90 min	phosphorylation of IKK-β↓ Jeong et al., 2014
	HEK293 cell	TNF-α	None	Ber	25 μmol/L	24 h	IKK-β gene expression↓ Pandey et al., 2008
	KM mice	HCM diet	Vehicle	Ber	50 mg/kg	2 w	IKKβ phosphorylation in liver and adipose tissue↓ Shang et al., 2010
	ARD Wistar rats	HCM diet	Normal diet	Ber	150 mg/kg/d	12 w	renal IKKβ protein level↓ Wan et al., 2013
	Fischer 344 rats	“Age”diet	Young rats	Bai	10,20 mg/kg/d	10 d	p-IKK expression↓ Kim et al., 2006
	HBE16 cells	LPS	Vehicle	Bai	10–100μM	24 h	p-IKK expression↓ Dong et al., 2015
	BALB/c mice	cisplatin	Vehicle	Bae	50 mg/kg/d	15 d	p-IKK protein expression↓ Sahu et al., 2015
	Raw264.7 cell	LPS	Vehicle	Rhe	17.5,35 μM	2 h	IKKβ activity↓ Gao et al., 2014
	BALB/c mice	LPS	CMCS	Rhe	20–80 mg/kg/d	7 d	p-IKKβ protein expression↓ Yu et al., 2015
IκBα	PM cell	LPS	Yohimbine	Ber	2 μM	90 min	phosphorylation of IκBα↓ Li et al., 2012
	Jurkat cell	TNF-α	None	Ber	50 μmol/L	18 h	IκB-α degradation↓ Pandey et al., 2008
	Mesangial cell	LPS	PDTC	Ber	30,90 μM	12 h	IκBα protein expression↑ Jiang et al., 2011
	BALB/c mice	DSS	CK	Ber	100 mg/kg	3 d	colon IκBα protein expression↑ Li et al., 2014
	C57BL/6 mice	LPS	Yohimbine	Ber	50 mg/mg	3 d	spleen IκBα phosphorylation↓ Li et al., 2012
	BALB/c mice	DSS	CK	Ber	100 mg/kg	3 d	p-IκBα protein expression of in cytoplasm of colon cell↓ Li et al., 2014
	Raw264.7 cell	LPS	BAY11-7082	Bae	10 μM	2 h	IκBα phosphorylation↓ Fan et al., 2013
	BALB/c mice	Cisplatin	Vehicle	Bae	50 mg/kg/d	15 d	p-IκBα protein expression↓ Sahu et al., 2015
	C57BL/6 mice	Surgery	SO	Bae	100 mg/kg/d	7 d	IκBα degradation↓ Wang W. et al., 2015
	WKY rats	LPS	SO	Bae	10 mg/kg	6 h	p- IκBα expression↓ Lee et al., 2011
	Microglial cells	OGD	Vehicle	Bai	40,20 ug/ml	4 h	p-IκBα protein level↓ Hou et al., 2012
	DBA/1 mice	CII	PBS	Emo	10 mg/kg	10 d	IκBα degradation↓ Hwang et al., 2013
	HUVECs	LPS	DMSO	Emo	10–50 μg/ml	30 min	IκBα degradation↓ Meng et al., 2010
	MEC	LPS	Vehicle	Emo	10,20,40 μg/ml	1 h	IκBα degradation↓ Yang Z. et al., 2014
	BMMCs	PMA+ A23187	PDTC	Emo	1–20 μM	1 h	p-IκBα / IκBα↓ Lu et al., 2013
	Raw264.7 cell	LPS	Vehicle	Rhe	17.5,35 μM	30 min	IκBα phosphorylation↓ Gao et al., 2014
	Chondrocytes	IL-1β	Vehicle	Rhe	10 μM	18 h	IκBα degradation↓ Domagala et al., 2006
	Raw264.7 cell	LPS	BAY11-7082	Aem	10,20 μM	12 h	IκBα degradation↓ Hu et al., 2014
	BALB/c mice	LPS	CMCS	Rhe	20–80 mg/kg/d	7 d	p-IκBα protein expression↓ Yu et al., 2015
NF-κB	PM	LPS	Yohimbine	Ber	2 μM	90 min	NF-κB translocation and phosphorylation↓ Li et al., 2012
	SD rats	Surgery	Interceed	Ber	0.75,1.5 mg/ml	14 d	NF-κB phosphorylation↓ Zhang et al., 2014
	SD diabets rats	STZ	Vehicle	Ber	200 mg/kg	12 w	renal NF-κB expression↓ Xie et al., 2013
	Jurkat cell	TNF-α	None	Ber	50 μmol/L	18 h	NF-κB activation↓ Pandey et al., 2008
	ARD wistar rats	HCM diet	Normal diet	Ber	150 mg/kg/d	12 w	Renal NF-κB DNA banding↓ Wan et al., 2013
	BALB/cN mice	Cisplatin	Vehicle	Ber	3 mg/kg	2 d	NF-κB expression↓ Domitrović et al., 2013
	C57BL/6 rats	Cigarettes	Vehicle	Ber	50 mg/kg	4d	lung NF-κB DNA banding↓ Lin K. et al., 2013
	BALB/c mice	LPS	Yohimbine	Ber	50 mg/Kg	3 d	ileum NF-κB activation↓ Li et al., 2011
	C3H/HeN, C3H/HeJ rats(colitis)	TNBS	Vehicle	Ber	10,20 mg/kg	3 d	colon NF-κB activation↓ Lee et al., 2010
	Raw264.7 cell	LPS	BAY11-7082	Bae	10 μM	2 h	NF-κB activation↓ Fan et al., 2013
	DBA/1 mice	CII	PBS	Emo	10 mg/kg	10 d	NF-κB binding activity↓ Hwang et al., 2013
	MEC	LPS	Vehicle	Emo	10,20,40 μg/m	1 h	NF-κB activation↓ Yang Z. et al., 2014
	SD rats	ADM	Benazepril	Rhe	100 mg/kg/d	6–12 w	Renal NF-κB activation↓ Ji et al., 2005
p65	PM cell	LPS	Mesalazine	Ber	10,20 μM	1 h	p65 phosphorylation↓ Jeong et al., 2014
	Jurkat cell	TNF-α	None	Ber	50 μmol/L	18 h	p65 phosphorylation and translocation↓ Pandey et al., 2008
	NIT-1 cell	LPS	Vehicle	Ber	2.5,5.0 μM	24 h	p65 phosphorylation↓ Hamsa and Kuttan, 2012
	Mesangial cell	LPS	PDTC	Ber	30,90 μM	12 h	p65 translocation↓ Jiang et al., 2011
	B16F-10 cell	LPS	Vehicle	LPS	2 μg/mL	2 h	p65 DNA-bound↓ Hamsa and Kuttan, 2012
	ARD Wistar rats	HCM diet	Normal diet	Ber	150 mg/kg/d	12 w	Renal p65 protein level↓ Wan et al., 2013
	C57BL/6 rats	Cigarettes	Vehicle	Ber	50 mg/kg	4 d	p65 translocation↓ Lin K. et al., 2013
	BALB/c mice	DSS	CK	Ber	100 mg/kg	3 d	p65 translocation↓ Li et al., 2014
	BALB/c mice	Cisplatin	Vehicle	Bae	50 mg/kg/d	15 d	p65 translocation↓ Sahu et al., 2015
	C57BL/6 mice	Surgery	SO	Bae	100 mg/kg/d	7 d	p65 expression↓ Wang W. et al., 2015
	C57BL/6 mice	Ang II	Vehicle	Bae	25 mg/kg	14 d	p65 expression↓ Wang A. W. et al., 2014
	Raw264.7 cell	LPS	BAY11-7082	Bae	10 μM	2 h	p65 translocation↓ Fan et al., 2013
	Cardiomyocytes	I/R	Vehicle	Bae	25 μM	30 min	p65 phosphorylation↓ Song et al., 2014
	ICR mice	Placebo	Ribavirin	Bai	375 mg/kg/d	7 d	p65 protein level↓ Wan et al., 2014
	WKY rats	LPS	SO	Bae	10 mg/kg	6 h	p-p65 expression↓ Lee et al., 2011
	DBA/1 mice	CII	PBS	Emo	10 mg/kg	10 d	p65 translocation↓ Hwang et al., 2013
	BALB/c mice	LPS	Saline	Emo	100 mg/kg/12h	3.5 d	p65 phosphorylation↓ Xiao et al., 2014
	Wistar rats	LPS	Vehicle	Emo	10 mg/kg/hr	1,2 h	p65 expression↓ Li A. et al., 2013
	HUVECs	LPS	IL-1β	Emo	10–50μg/ml	30 min	p65 translocation↓ Meng et al., 2010
	MEC	LPS	GW9662	Emo	10,20,40 μg/ml	1 h	p-p65 expression↓ Yang Z. et al., 2014
	MDA-MB-435s	TNF-α	Vehicle	Rhe	50–200 μM	48 h	p65 nuclear translocation↓ Fernand et al., 2011
	Raw264.7 cell	LPS	Vehicle	Rhe	17.5,35 μM	1 h	p65 level in nuclear↓ Gao et al., 2014
	BALB/c mice	LPS	CMCS	Rhe	20–80 mg/kg/d	7 d	p-p65 protein expression↓ Yu et al., 2015
p50	B16F-10 cell	LPS	Vehicle	Ber	2 μg/mL	2 h	p50 DNA-bound↓ Hamsa and Kuttan, 2012
	ARD Wistar rats	HCM diet	Normal diet	Ber	150 mg/kg/d	12 w	renal p50 protein level↓ Wan et al., 2013
	DBA/1 mice	CII	PBS	Emo	10 mg/kg	10 d	p50 translocation↓ Hwang et al., 2013
	MDA-MB-435s	TNF-α	Vehicle	Rhe	50–200 μM	48 h	p50 nuclear translocation↓ Fernand et al., 2011
GSK3β	HT-29/B6 cell	TNF-α	BAY11-7082, Genistein	Ber	50 μM	26,2 h	GSK3β phosphorylation↓ Amasheh et al., 2010
IRF3	PM	LPS	Yohimbine	Ber	2 μM	2 h	IRF3 phosphorylation↓ Li et al., 2012
	BALB/c mice	LPS	Yohimbine	Ber	50 mg/kg	3 d	spleen IRF3 phosphorylation↓ Li et al., 2012
	DC1.2 cell	Poly(I:C)	Vehicle	Rhe	1–10 μM	5 h	p-IRF3 expression↓ Yuan et al., 2015

It has been identified that, MyD88 is recruited by TLR4 at plasma membrane to stimulate the initial activation of IKK, and it may be responsible for the early peak in NF-κB activity (Cheng Z. et al., 2015). Apart from MyD88, there are many other adapter molecules (such as TRAF3, TRAM and TRADD) sharing similar activity. NIK will promote NF-κB activation once combined with TRAF2 (Lee et al., 2014). Among them, MyD88 has been most systemically studied both *in vivo* and *in vitro*. In respect of these adaptor molecules, Ber and Bai negatively regulate their protein expressions (Pandey et al., 2008; Hou et al., 2012; Lim et al., 2012; Chen C. C. et al., 2014; Feng et al., 2014; Wan et al., 2014), however the main constituents from RR are rarely mentioned.

**Figure 1:**
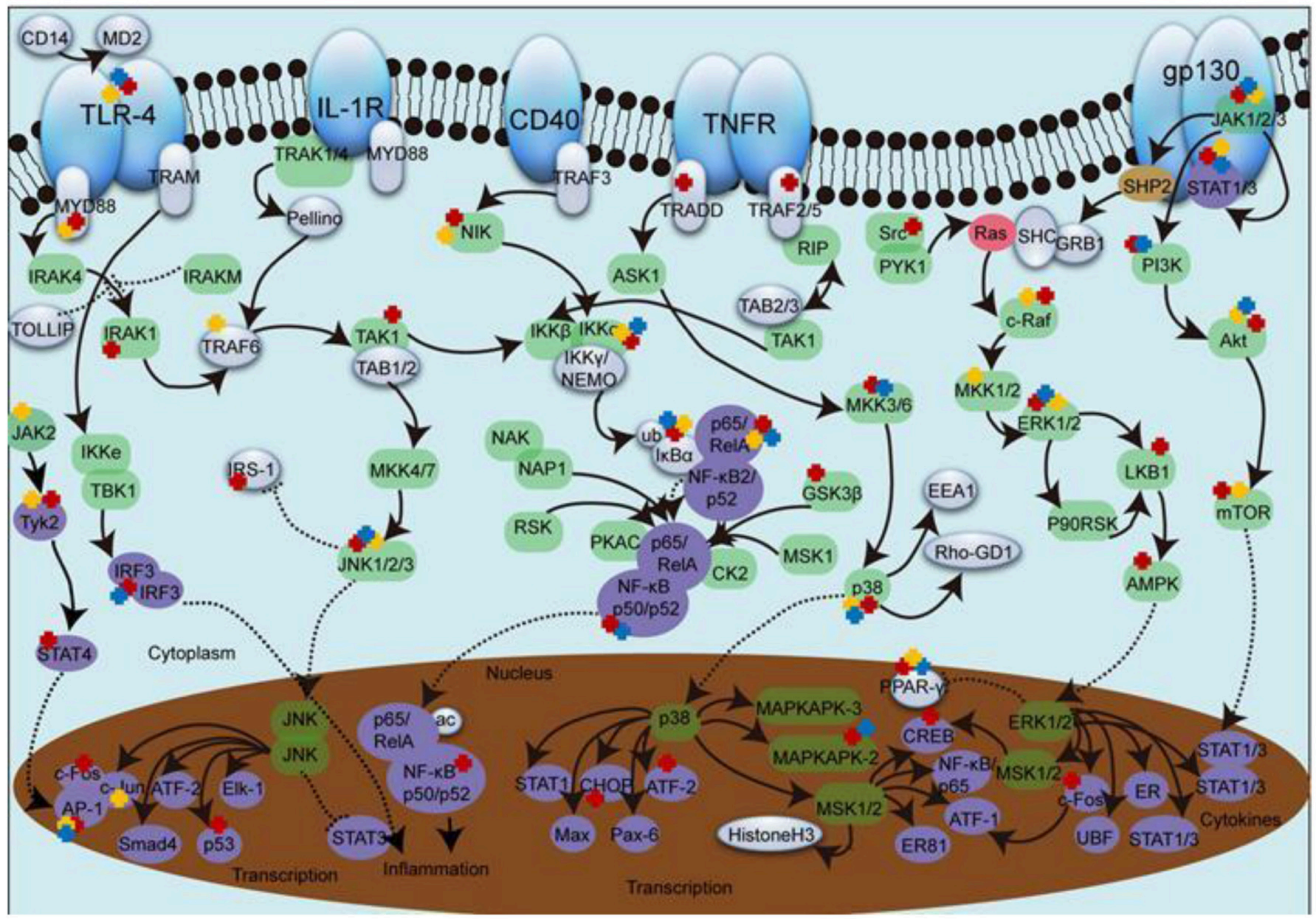
**The effect of SHXXT alkaloids on inflammatory pathway molecules**. 1. The green ellipse represents kinase. 2. The purple ellipse represents transcription factor. 3. The red ellipse represents GTpase. 4. The brown ellipse represents phosphatase. 5. The solid arrow represents direct stimulatory modification. 6. The dotted arrow represents translocation. 7. The dotted “T” represents direct inhibitory modification. 8. The red, yellow and blue cross represents the target influenced by RC, RS, and RR constituents, respectively.

Enzyme complex IKK (α-γ) have a crucial role in regulating NF-κB signaling pathway (Bagnéris et al., 2015). In general, IκBα forms a heterodimer with p65 (RELA) and p50 (NF-κb1), making NF-κB sequestered in cytoplasm. Once activated, IκBα goes phosphorylated meanwhile p65 is liberated and translocate into nuclear, which leads to gene transcription (Pandey et al., 2008). Depicted in Figure 1, the majority of current studies focus on upstream molecules from IKK to p65. Data in Table 1 show the main constituents of SHXXT can inhibit (1) the expression and phosphorylation of IKK, (2) the expression, phosphorylation and degradation of IκBα, (3) the expression, phosphorylation and translocation of p65 and (4) the expression, phosphorylation, DNA banding and activation of NF-κB in multiple *in-vivo* and *in-vitro* models, such as mesangial (Jiang et al., 2011), RAW264.7 (Fan et al., 2013), MEC (Yang Z. et al., 2014) etc., and ARD rats (Wan et al., 2013), C57BL/6 mice (Wang W. et al., 2015), DBA/1 mice (Hwang et al., 2013), etc.

We know that, GSK3β is not active until dephosphorylated, and the activation will promote inflammation process undergoes Alzheimer Disease and diabetes (Venna et al., 2015). IRF3 is a target of TLR-4 signaling pathway, acting as regulating and activating the transcription of interferon which results inflammatory responses (Cheng B. C. Y. et al., 2015). Briefly, phosphorylation of these two downstream molecules are both identified to be reversed by Ber or Rhe treatment in either animal or cell inflammatory model (Amasheh et al., 2010; Li et al., 2012; Yuan et al., 2015), which cover the effect shortage of RS constituents at this part.

### Effect of the active constituents on molecules in MAPK pathway

MAPK can be divided into several subfamilies including p38, ERK and JNK (Lou et al., 2011). Upstream TAK1 forms a complex consist of TAB1, TAB2, and TRAF6 and then sequentially activate MKK and JNK. The presence of Ras will activate c-raf, MEK and ERK, followed by c-fos regulation once transported into nucleus. Subsequently, the regulated c-fos recruits c-jun to form AP-1 complex (Figure 1).

Accumulative data shown in Table 2 leads to a conclusion that p38, ERK, and JNK attract the most focus of study in MAPK pathway. *In-vitro* study results reveal that the increased level of p38, ERK or JNK phosphorylation stimulated by cytokines/chemokines like LPS (Lin Y. et al., 2013), IL-1β (Legendre et al., 2007), oxLDL (Chen J. et al., 2014), PMA (Huang Z. et al., 2011), ischemia (Song et al., 2014), OGD (Hou et al., 2012), HG (Li et al., 2009) and CoCl_2_ (Fernand et al., 2011), or *in-vivo* elevated level induced by insulin (Lu et al., 2010), collagen (Wang Z. et al., 2014) and cisplatin (Sahu et al., 2015) can be significantly attenuated by either RR, RC, or RS constituent intervention. To further investigate whether p38, ERK and JNK are the only targets, molecules lied on the upstream and downstream are taken into consideration. Turns out, Ber, Bai as well as Rhe treatments all show inhibitory effect on MEK phosphorylation (Shen et al., 2011; Lim et al., 2012; Liu et al., 2015). Nevertheless, for the enhanced phosphorylation of TAK1, Ber is the only reported SHXXT constituent (Zhang et al., 2014). In addition, Ber, Bai, or Rhe also display markedly suppressing effect on endonuclear translocation factors like c-fos and CREB (Hamsa and Kuttan, 2012), c-jun (Hou et al., 2012), ATF-2(Legendre et al., 2007), CHOP (Zha et al., 2010), or AP-1 complex (Domagala et al., 2006).

**Table 2:** **Effect of the active constituents on molecules in MAPK pathway**.

**Target**	**Animal or cell culture**	**Model building**	**Control (P or N)**	**Drug**	**Dose**	**Treat time**	**Result**
MEK	Fischer 344 rats	“Age” diet	Young rats	Bai	10,20 mg/kg/d	10 d	MEK phosphorylation↓ Kim et al., 2006
	VSMC	PDGF	Vehicle	Bai	5–40 μM	48 h	p-MEK phosphorylation↓ Hu et al., 2010
	U251/U87 cell	None	Vehicle	Ber	15 μM	1–7 d	p-MEK phosphorylation↓ Liu et al., 2015
	Jurkat cell	SDF-1β	Pyscion	Emo	1 μg/ml	1 h	p-MEK phosphorylation↓ Shen et al., 2011
TAK1	SD rats	Surgery	Interceed	Ber	0.75,1.5 mg/ml	14 d	TAK phosphorylation Zhang et al., 2014
JNK	THP-1 cell	oxLDL	Vehicle	Ber	25 μM	1 h	JNK phosphorylation↓ Chen J. et al., 2014
	RAW264.7 cell, PM	LPS	Vehicle	Ber	5 μM	2 h	JNK phosphorylation↓ Jeong et al., 2009
	PM	LPS	Yohimbine	Ber	2 μM	90 min	JNK activation↓ Li et al., 2012
	CIA SD rats	Collagen	PBS	Ber	200 mg/kg	28 d	JNK expression↓ Wang Z. et al., 2014
	SD rats	Surgery	Interceed	Ber	0.75,1.5 mg/ml	14 d	JNK phosphorylation↓ Zhang et al., 2014
	BALB/c mice	LPS	Yohimbine	Ber	50 mg/kg	3 d	Spleen JNK phosphorylation↓ Li et al., 2012
	CIA SD rats	Collagen	PBS	Ber	200 mg/kg	28 d	p-JNK expression↓ Wang Z. et al., 2014
	NIT-1 cell	LPS	Vehicle	Ber	2.5,5.0 μM	24 h	p-JNK expression↓ Hamsa and Kuttan, 2012
	Cardiomyocytes	I/R	Vehicle	Bae	25 μM	30 min	JNK1/2 phosphorylation↓ Song et al., 2014
	Microglial cells	OGD	Vehicle	Bai	40,20 ug/ml	4 h	p-JNK protein level↓ Hou et al., 2012
	BALB/c mice	Cisplatin	Vehicle	Bae	50 mg/kg/d	15 d	p-JNK expression↓ Sahu et al., 2015
	C57BL/6 mice	Surgery	SO	Bae	100 mg/kg/d	7 d	p-JNK expression↓ Wang W. et al., 2015
	TRMs rats	None	WT	Bae	10–40 mg/kg	14 d	p-JNK expression↓ Mao et al., 2014
	SAP SD rats	ST	SO	Emo	30 mg/kg	6 h	p-JNK protein expression↓ Wu et al., 2013
	BMMCs	PMA+ A23187	SP600125	Emo	1–20 μM	1 h	p-JNK/JNK↓ Lu et al., 2013
	MEC	LPS	Vehicle	Emo	10,20,40 μg/ml	1 h	p-JNK expression↓ Yang Z. et al., 2014
	Chondrocytes	IL-1β	DMSO	Rhe	100 μM	18 h	JNK activation↓ Legendre et al., 2007
	Raw264.7 cell	LPS	SP600125	Aem	5,10,20 μM	4 h	JNK phosphorylation↓ Hu et al., 2014
ERK	PM	LPS	Yohimbine	Ber	2 μM	90 min	ERK activation↓ Li et al., 2012
	HepG2 cell	Palmitate	PD98059	Ber	10 μM	30 min	ERK phosphorylation↓ Lu et al., 2010
	BV2 microglial	IFN-γ	Vehicle	Ber	10 μM	30 min	ERK phosphorylation↓ Lu et al., 2010
	RAW264.7 cell, PM	LPS	Vehicle	Ber	5 μM	2 h	ERK phosphorylation↓ Jeong et al., 2009
	BALB/c mice	LPS	Yohimbine	Ber	50 mg/kg	3 d	Spleen ERK phosphorylation↓ Li et al., 2012
	CIA SD rats	Collagen	PBS	Ber	200 mg/kg	28 d	p-ERK expression↓ Wang Z. et al., 2014
	U266 cells	IL-6	PD98059	Bae	50 μM	1 h	ERK1/2 phosphorylation↓ Liu et al., 2010
	Fischer 344 rats	“Age” diet	Young rats	Bai	10,20 mg/kg/d	10 d	p-ERK1/2 expression↓ Kim et al., 2006
	BALB/c mice	Cisplatin	Vehicle	Bae	50 mg/kg/d	15 d	p-ERK expression↓ Sahu et al., 2015
	C57BL/6 mice	Surgery	SO	Bae	100 mg/kg/d	7 d	p-ERK expression↓ Wang W. et al., 2015
	C57BL/6 mice	Ang II	Vehicle	Bae	25 mg/kg	14 d	p-ERK1/2 expression↓ Wang A. W. et al., 2014
	Chondrocytes	IL-1β	Vehicle	Rhe	10 μM	18 h	ERK1/2 phosphorylation↓ Domagala et al., 2006
	Chondrocytes	IL-1β	DMSO	Rhe	100 μM	18 h	ERK activation↓ Legendre et al., 2007
	BALB/c mice	LPS	Vehicle	Emo	1–4 mg/kg	12 h	ERK phosphorylation↓ Li D. et al., 2013
	MEC	LPS	Vehicle	Emo	10,20,40 μg/ml	1 h	p- ERK expression↓ Yang Z. et al., 2014
	BMMCs	PMA+ A23187	U0126	Emo	1–20 μM	1 h	p- ERK / ERK ↓ Lu et al., 2013
	Raw264.7 cell	LPS	PD98059	Aem	5,10,20 μM	12 h	ERK1/2 phosphorylation↓ Hu et al., 2014
P38	THP-1	oxLDL	Vehicle	Ber	25 μM	1 h	p38 phosphorylation↓ Chen J. et al., 2014
	THP-1	PMA	Vehicle	Ber	5–50 μM	1 h	Block p38 pathway Huang Z. et al., 2011
	RAW264.7 cell, PM	LPS	Vehicle	Ber	5 μM	2 h	p38 phosphorylation↓ Jeong et al., 2009
	CIA SD rats	Collagen	PBS	Ber	200 mg/kg	28 d	p-p38 expression↓ Wang Z. et al., 2014
	SD rats	LPS	Vehicle	Ber	100 mg/kg	24 h	p38 expression↓ Godugu et al., 2014
	BALB/cN mice	Cisplatin	Vehicle	Ber	3 mg/kg	2 d	Renal p38 expression↓ Domitrović et al., 2013
	Cardiomyocytes	I/R	Vehicle	Bae	25 μM	30 min	p38 phosphorylation↓ Song et al., 2014
	Microglial cells	OGD	Vehicle	Bai	40, 20 ug/ml	4 h	p-p38 protein level↓ Hou et al., 2012
	BALB/c mice	Cisplatin	Vehicle	Bae	50 mg/kg/d	15 d	p-p38 expression↓ Sahu et al., 2015
	C57BL/6 mice	Surgery	SO	Bae	100 mg/kg/d	7 d	p-p38 expression↓ Wang W. et al., 2015
	TRMs rats	None	WT	Bae	10–40 mg/kg	14 d	p-p38 expression↓ Mao et al., 2014
	HUVECs	LPS	Vehicle	Rhe	0,5,10,20 μM	24 h	p38 phosphorylation↓ Hu et al., 2013
	HUVECs	LPS	ip38	Rhe	20 μM	24 h	p38 phosphorylation↓ Lin Y. et al., 2013
	SAP SD rats	ST	SO	Emo	30 mg/kg	6 h	p-p38 protein expression↓ Wu et al., 2013
	HBZY-1	HG	SB203580	Emo	30–60 μM	24 h	p-p38 protein expression↓ Li et al., 2009
	MEC	LPS	Vehicle	Emo	10,20,40 μg/ml	1 h	p-p38 protein expression↓ Yang Z. et al., 2014
	BMMCs	PMA+ A23187	SB203580	Emo	1–20 μM	1 h	p-p38 /p38↓ Lu et al., 2013
	HUVECs	CoCl_2_	Vehicle	Rhe	50 μM	6 h	p-ERK acivation↓ Fernand et al., 2011
	Raw264.7 cell	LPS	SB203580	Aem	10,20 μM	4 h	p38 phosphorylation↓ Hu et al., 2014
IRS-1	3T3-L1 cell	TNF-α	Pioglitazone	WE	30–100 mg/L	24 h	IRS-1 phosphorylation↓ Yuan et al., 2014
	HepG2 cell	Palmitate	SS	Ber	0.1–10 μM	30 min	IRS-1 phosphorylation↓ Lou et al., 2011
MAPK APK2	HUVECs	LPS	ip38	Rhe	20 μM	24 h	MAPKAPK2 phosphorylation↓ Lin Y. et al., 2013
CREB	B16F-10 cell	LPS	Vehicle	Ber	2 μg/ml	2 h	CREB DNA-bound↓ Hamsa and Kuttan, 2012
c-Rel	B16F-10 cell	LPS	Vehicle	Ber	2 μg/ml	2 h	c-Rel DNA-bound↓ Hamsa and Kuttan, 2012
c-fos	B16F-10 cell	LPS	Vehicle	Ber	2 μg/ml	2 h	c-Fos DNA-bound↓ Hamsa and Kuttan, 2012
c-jun	Microglial cells	OGD	Vehicle	Bai	40 ug/ml	4 h	p-c-jun protein level↓ Hou et al., 2012
AP-1	Chondrocytes	IL-1β	DMSO	Rhe	100 μM	18 h	AP-1 DNA binding↓ Legendre et al., 2007
	Chondrocytes	IL-1β	Vehicle	Rhe	10 μM	18 h	AP-1 DNA binding↓ Domagala et al., 2006
	ICR mice	Placebo	Ribavirin	Bai	375 mg/kg/d	7 d	c-jun/AP-1 expression↓ Wan et al., 2014
ATF2	B16F-10 cell	LPS	Vehicle	Ber	2 μg/ml	2 h	ATF-2 DNA-bound↓ Hamsa and Kuttan, 2012
CHOP	J744A.1 macrophages	Protease inhibitor	Vehicle	Ber	0–2.0 mg/ml	2 h	nuclear CHOP expression↓ Zha et al., 2010

### Effect of the active constituents on molecules in AMPK pathway

AMPK serves as a cellular energy sensor to modulate lipid metabolism, and it can be activated by upstream kinases like LKB1 and CaMKK (Yang Y. et al., 2014; Li N. S. et al., 2016). There is a mechanism underlined the relationship, thus once AMPK activated, the nuclear translocation of Nrf2 is promoted, which contribute to the diminution of pro-inflammatory cytokines production. Nrf2 can also drive downstream HO-1 expression in with the considerable beneficial protect effect against cell injury from inflammatory response like diabetes mellitus (Agca et al., 2014). PPAR-γ is identified as a primary regulator of gene expression for inflammation and a pharmacological receptor of insulin-sensitizing drugs (Choi et al., 2014).

As summed up in Table 3, the current study status demonstrate that Ber from RC exert the most comprehensive effect compared with other constituents form RR and RS, pathway molecules from upstream to downstream, including CaMKII, LKB1, PPAR-γ (Legendre et al., 2007), AMPK (Lu et al., 2010), Nrf2 and HO-1(Mo et al., 2014) are all verified to be the effective targets of Ber. In addition, Emo (Yang Z. et al., 2014; Wang T. et al., 2015), Bai and Bae (Lim et al., 2012; Ma et al., 2012; Feng et al., 2013; Tsai et al., 2014) as well affect some of those molecules. Given this investigation situation, it seems that constituents from either RR, RS, or RC can block AMPK pathway by cross-talk regulating pathway molecules.

**Table 3:** **Effect of the active constituents on molecules in AMPK pathway**.

**Target**	**Animal or cell culture**	**Model building**	**Control (P or N)**	**Drug**	**Dose**	**Treat time**	**Result**
CaMK-II	BV2 microglial cell	LPS or IFN-γ	Vehicle	Ber	10 μM	2 h	CaMKII _(Thr286)_ phosphorylation↑ Lu et al., 2010
LKB1	BV2 microglial cell	LPS or IFN-γ	Vehicle	Ber	10 μM	2 h	LKB1 phosphorylation↑ Lu et al., 2010
AMPK	BV2 microglial cell	LPS or IFN-γ	Vehicle	Ber	10 μM	2 h	AMPK _(*Thr*172)_ phosphorylation↑ Lu et al., 2010
	Hela cell	None	Compound C	Bai	1 μM	3 h	AMPK phosphorylation↑ Ma et al., 2012
HO-1	PM	LPS	Vector	Ber	10 μM	24 h	HO-1 mRNA expression↑ Mo et al., 2014
	SD rats	LPS	Vehicle	Bae	20 mg/kg	7 h	HO-1 protein expression↑ Tsai et al., 2014
	BALB/c mice	Dox	Vehicle	Bae	25 mg/kg	24 d	HO-1 protein expression↑ Sahu et al., 2016
	C57BL/6 mice	OVA	Dex	Emo	10 mg/kg	3 d	HO-1 mRNA expression↑ Wang T. et al., 2015
Nrf2	PM	LPS	Vector	Ber	10 μM	24 h	Nrf2 translocation↑ Mo et al., 2014
	SD rats	LPS	Vehicle	Bae	20 mg/kg	7 h	Nrf2 nuclear translocation↑ Tsai et al., 2014
	BALB/c mice	Dox	Vehicle	Bae	25 mg/kg	24 d	Nrf2 protein expression↑ Sahu et al., 2016
PPAR -γ	3T3-L1 cell	TNF-a	Pioglitazone	WE	30 mg/L	24 h	PPAR-γ mRNA expression↑Yuan et al., 2014
	SD rats	LPS	SR-202	Bai	25 mg/kg	3 d	intestinal PPAR-γ level↓ Feng et al., 2013
	Fischer 344 rats	Aged	TZD;GW9662	Bai	10 mg/kg	3 d	PPAR-γ protein expression↓ Lim et al., 2012
	HBZY-1	HG	SB203580	Emo	30–60 μM	24 h	PPAR-γ protein expression↑ Li et al., 2009
	MEC	LPS	Rosiglitazone	Emo	10 μg/ml	1 h	PPAR-γ activation↑ Yang Z. et al., 2014

### Effect of the active constituents on molecules in JAK/STAT pathway

The activation of JAK catalyze Tyr phosphorylation so that STAT can be combined with receptor protein, then transported into nucleus to regulate transcription. It has been reported that STAT1 and STAT5, the downstream molecules of IFN-γ, are also likely to be implicated in inflammation (Chmielewski et al., 2015; Li X. et al., 2015). Akt functions as emerging crucial regulator of multiple cellular processes, such as apoptosis, differentiation, survival, etc., (Piao et al., 2015). Moreover, recent studies indicate PI3K/Akt can lead to an elevated expression level of COX-2 and iNOs in inflammatory macrophages (Liou et al., 2014). Further activated mTOR can regulate cell growth, differentiation as well as transcription and it tends to perform abnormally in diabetes models (Hua and Hu, 2015).

For JAK/STAT pathway, constituents from RC, RS and RR are all showing inhibitory activity, typical targets include JAK (Kim et al., 2011; Qi et al., 2013; Subramaniam et al., 2013), STAT (Cui et al., 2009; Liu et al., 2010; Kim et al., 2015) and Akt (Lou et al., 2011; Hu et al., 2014; Wang A. W. et al., 2014), all of which are proved to be influenced by Ber, Bae, Bae, Emo or Aem in either *in-vivo* or *in-vitro* models (Table 4). On the other hand, results in the study concerning about downstream molecular present main RR constituent's effect-weakness on targets like Tyk2. Apparently, RS and RC cover the shortfalls of RR's poor activity in downstream pathway, which partly supports the synergistic theory of drug combination aiming at promoting curative effect.

**Table 4:** **Effect of the active constituents on molecules in JAK/STAT pathway**.

**Target**	**Animal or cell culture**	**Model building**	**Control (P or N)**	**Drug**	**Dose**	**Treat time**	**Result**
JAK1	Raw264.7 cell	LPS	Vehicle	Bae	20–80 μM	2 h	JAK1 phosphorylation↓ Qi et al., 2013
	NOP2 cells	IL-6	None	Bae	50 μM	1 h	JAK1 phosphorylation↓ Liu et al., 2010
JAK2	Raw264.7 cell	LPS	Vehicle	Bae	20–80 μM	2 h	JAK2 phosphorylation↓ Qi et al., 2013
	HepG2	None	Vehicle	Emo	50 μM	12 h	JAK2 phosphorylation↓ Subramaniam et al., 2013
JAK3	Nb2 cell	IL-2	Vehicle	Ber	1–10 μM	1 h	JAK3 phosphorylation↓ Kim et al., 2011
STAT1	NOD rats CD4+ T cell	None	Vehicle	Ber	200 mg/kg 5, 10 μM	2 w	STAT1 phosphorylation↓ Cui et al., 2009
	BALB/c mice	LPS	Yohimbine	Ber	50 mg/kg	3 d	Spleen STAT1 phosphorylation↓ Li et al., 2012
	U266 cells	IL-6	None	Bae	12.5–50 μM	1 h	STAT1 phosphorylation↓ Liu et al., 2010
STAT3	NOD rats CD4+ T cell	None	Vehicle	Ber	200 mg/kg 5, 10 μM	2 w	STAT3 phosphorylation↓ Cui et al., 2009
	U266cells	IL-6	None	Bae	50,100 μM	1 h	STAT3 phosphorylation↓ Liu et al., 2010
	GSCs	None	Vehicle	Emo	5 μM	24 h	p-STAT3 phosphorylation↓ Kim et al., 2015
	RPMI8266	IL-6	Dox	Emo	50 μmol/L	12 h	STAT3 phosphorylation↓ Muto et al., 2007
STAT5	Nb2 cell	IL-2	Vehicle	Ber	1,3,7,10 μM	1 h	STAT5 phosphorylation↓ Kim et al., 2011
STAT4	NOD rats CD4+ T cell	None	Vehicle	Ber	200 mg/kg 5, 10μM	2 w	STAT4 phosphorylation↓ Cui et al., 2009
	Arthritis mice	kaolin	Prednisolone	Ber	10–50 mg/kg	6 d	synovial expression STAT4↓ Kim et al., 2011
STAT6	Arthritis mice	kaolin	Prednisolone	Ber	10–50 mg/kg	6 d	synovial expression STAT6↓ Kim et al., 2011
Tyk2	BALB/c mice	LPS	Yohimbine	Ber	50 mg/kg	3 d	Spleen Tyk2 phosphorylation↓ Li et al., 2012
	NOP2 cells	IL-6	None	Bae	25 μM	1 h	Tyk2 phosphorylation↓ Liu et al., 2010
Src-P	HT-29/B6 cell	TNF-α	BAY11-7082, Genistein	Ber	50 μM	26 h	Src-P phosphorylation↓ Amasheh et al., 2010
Akt	HT-29/B6 cell	TNF-α	BAY11-7082, Genistein	Ber	50 μM	26 h	Akt phosphorylation↓ Amasheh et al., 2010
	HepG2 cell	Paimitate	PD98059,SS, BAY11-7082	Ber	0.1–10 μM	30 min	Akt phosphorylation↓ Lou et al., 2011
	NOP2 cells	IGF-1	None	Bae	10 μM	30 min	Akt phosphorylation↓ Liu et al., 2010
	HUVECs	CoCl_2_	Vehicle	Rhe	50 μM	6 h	p-Akt activation↓ Fernand et al., 2011
	Raw264.7 cell	LPS	LY294002	Aem	10,20 μM	4 h	Akt phosphorylation↓ Hu et al., 2014
	C57BL/6 mice	Ang II	Vehicle	Bae	25 mg/kg	14 d	p-Akt expression↓ Wang A. W. et al., 2014
	SD rats	Adjuvant	Ibuprofen	Rhe	50 mg/kg	21 d	p-Akt/Akt level↓ Cong et al., 2012
PI3K	HUVECs	CoCl_2_	Vehicle	Rhe	50 μM	6 h	PI3K activation↓ Fernand et al., 2011
	HT-29/B6 cell	TNF-α	BAY11-7082, Genistein	Ber	50 μM	26 h	PI3K activation↓ Amasheh et al., 2010
mTOR	CRC cells	None	Vehicle	Ber	15–60 μM	24 h	mTOR phosphorylation↓ Li W. et al., 2015
	C57BL/6 mice	Ang II	Vehicle	Bae	25 mg/kg	14 d	p-mTOR expression↓ Wang A. W. et al., 2014

## Pharmacokinetic level

Traditional Chinese medicines are frequently orally administrated and the absorption of active constituents are confirmed to be influenced by efflux pumps and intestinal transporters (ITs) (Park et al., 2012; Zumdick et al., 2012). In general, ITs widely distribute in intestinal membrane and can be divided into two categories. One accounts for external substance's intracellular transport, such as OCTs and SGLT1 (Moran et al., 2014; Couroussé and Gautron, 2015). The other one, like P-gp, MRP and BCRP, is functioning as efflux pump to make drug or toxin back to lumen (Yamagata et al., 2007; Juan et al., 2010; Zeng et al., 2015). There are many isolate reports showing SHXXT's main constituents have an unexpectedly low concentration in plasma with oral administration, making it challenged to explain its positive effects in inflammatory therapies.

*In-vitro* research on the efflux pump and ITs normally use Caco-2 cell or MDCK cell for they both have similar structure of differential intestinal epithelial cell with apical side and basolateral side (Chen et al., 2013; Schexnayder and Stratford, 2015; Obringer et al., 2016). Currently, it is verified that Bai from RS is the substrate of both MRP2 and BCRP (Kalapos-Kovács et al., 2015), and another RS constituent Bae is also pumped out by MRP (Zhang et al., 2007). Rhe, Emo and Aem from RR are substrate of BCRP, MRP and P-gp respectively (Wang J. et al., 2011; Liu et al., 2012; Ye et al., 2013), those ITs at least partly reduce the bioavailability of SHXXT constituents by diminishing their intracellular transport. Similarly, the absorption of Ber, Pal, Cop and Jat form RC is reported to be promoted by OCTs while inhibited by P-gp (Chen et al., 2008; Zhang et al., 2011; Sun et al., 2014). In addition to OCTs, SGLT1 also contributes to uptake (Zhang et al., 2012). Thus, any constituents in SHXXT which suppress the MRP2, BCRP, and P-gp activation or, on the other hand, up-regulate OCTs and SGLT1 activation may be considered to exert mutual reinforcement property by promoting bioavailability.

**Figure 2:**
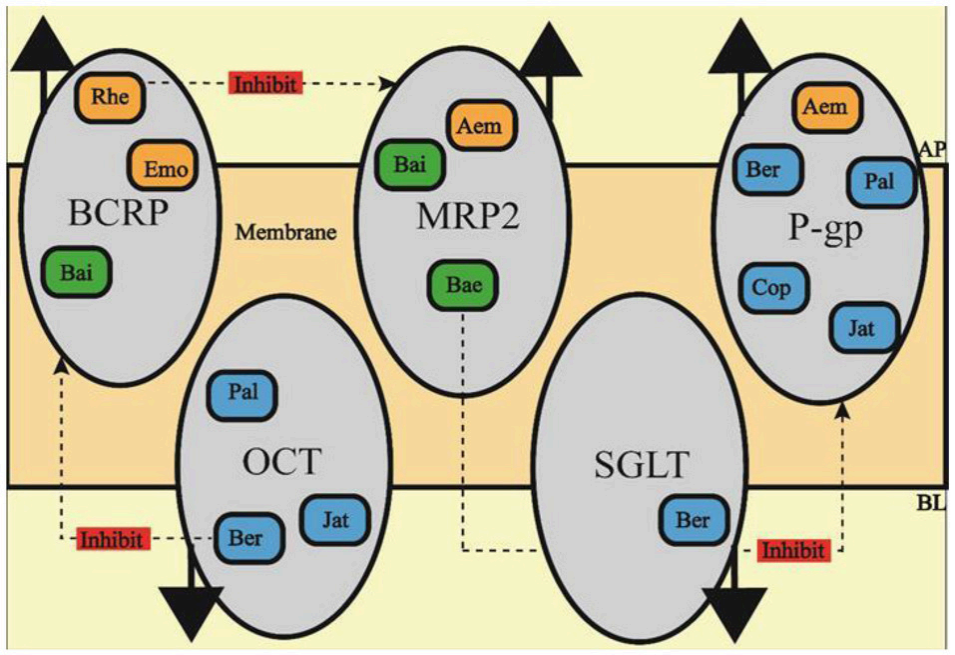
**The effect of SHXXT constituents on ITs**. Ber, berberine; Cop, coptisine; Pal, palmatine; Jat, jatrorrhizine; Bai, baicalin; Bae, baicalein; Emo, emodin; Aem, aloe-emodin; Rhe, rhein.

In return, constituents in SHXXT show retroaction on those efflux pump or ITs. Depicted in Figure 2, Firstly, P-gp, which reduces the absorption of Ber, Pal, Cop, Jar, and Aem, is proved to be inhibited by Bae treatment (Cho et al., 2011). Secondly, Rhe can suppress MRP's activation which may lead the increasing uptake of Aem, Bai, and Bae (Shia et al., 2013). Last but not least, Ber can as well decrease BCRP activation, which is capable of promoting the intracellular concentrations of Bai, Emo, and Rhe (Tan et al., 2013).

## Discussion

TCM normally used as prescription so as to recruit active contents from different herbals. Modern mutual reinforcement theory believes pharmacodynamics effect after herbal combination is not simply equal to the summing up of each herbal, but to a certain extent, should be more than that. Under most circumstances, a prescription can bring out more advantages in regards of safety and efficacy aspects than a single herb does (Song et al., 2013). Apart from expanding effect on one specific part, the combination of several herbals can also give rise to respective effect on different parts, which in other words, supplement other herbals' disadvantages or helping other herbals to perform their property in a better way.

Inflammatory signal transduction is quite complex network, and suppression on any intersection can partly contribute to the prevention of inflammation process. SHXXT have been high-lighted based on their widely appearance in inflammation-associated treatment for centuries. Clinically, SHXXT is a preferred drug for “coexistence of cold and heat” (Zhang et al., 2013c). With the constantly deepen researches, it is widely used in the treatment for anti-pathogens, anti-inflammation, gastric mucosal protection, hemostasis, anti-diabetes and so on (Li and Guo, 2010). As depicted in Figure 1, target with three colored “cross” is to be influenced by constituents form all three compositions (TLR-4, ERK, JNK, p38, Akt, etc.), which lead a fold increase of the final effect. On the other hand, target with less than three “cross” suggest at least one composition was not valid at this part. For example,

1. Ber from RC is reported to affect TAK1 and interaction between LPS and TLR-4, while RR and main RS constituents barely mentioned.
2. Bae from RS and Ber from RC inhibit Tyk2 phosphorylation, while no main RR constituent has similar effect.
3. Rhe from RR and Ber from RC reduce IRF3 phosphorylation level, while the effect of main constituent of RS isn't that clear, etc.

The connotative meaning of synergism at pharmacodynamics level is to enhance the effect on a certain target, as well as to expand target-affecting scope, just like what SHXXT constituents have performed. As for the pharmacokinetic level, shown in Figure 2, Ber form RS, Rhe form RR and Bae form RC is capable of improving the uptake or reducing the efflux of constituents from the other composition, which ultimately reaches the goal of synergistically influence inflammatory processes and eventually make this formula's anti-inflammatory action stronger and wider.

Nowadays, elevated attention has been paid to dose-effect relationship. There is a complicated process which can be expressed as “theory-methodology-formulation-medication-dosage” in TCM clinical therapeutics, showing how important for a formula prescription to have a specific herbal dosage (Zha et al., 2015). Basically for western medicine, these is a positive correlation between dose and toxicity. However, TCM at a large dosage tends to have good therapy efficacy with slight side effect (Wang et al., 1983). The dosage of Chinese herbals in clinical cases or experimental studies is usually at a relatively higher level than that documented in ancient TCM records (Peng, 2003; Sun, 2007). RR as an example, the dosage to treat cholestasis in clinical is more than four times the regular dose recommended in the Chinese pharmacopeia (Zhang et al., 2016). For now, the widespread explanation is that drug should be administrated to the patient with the correct disorder indications, otherwise it will produce dosage variety and individual detrimental effect (Zhao et al., 2015). As displayed in Table 5, dosage of constituents from SHXXT has a big range with no obvious rule to follow, it is possibly due to different tested animals or cells may have different drug sensitivities, but still need further clarification.

**Table 5:** **The dose range of SHXXT constituent used *in-vivo* and *in-vitro***.

**Constituent**	**Model**	**Dose lower limit**	**Dose upper limit**
Ber	Cells	0.1 μM Lou et al., 2011	90μM Jiang et al., 2011
	Mice	3 mg/kg Domitrović et al., 2013	50mg/kg Li et al., 2011
	Rats	50 mg/kg Li et al., 2012	200 mg/kg Muto et al., 2007
Bai	Cells	1 μM Ma et al., 2012	100 μM Dong et al., 2015
	Mice	100 mg/kg Feng et al., 2014	375 mg/kg Wan et al., 2014
	Rats	10 mg/kg Lim et al., 2012	25 mg/kg Feng et al., 2013
Bae	Cells	10 μM Fan et al., 2013	80 μM Qi et al., 2013
	Mice	25 mg/kg Sahu et al., 2016	100 mg/kg Wang W. et al., 2015
	Rats	10 mg/kg Lee et al., 2011	40 mg/kg Mao et al., 2014
Emo	Cells	1 μM Lu et al., 2013	182.5 μM Meng et al., 2010
	Mice	1 mg/kg Li D. et al., 2013	100 mg/kg Xiao et al., 2014
	Rats	10 mg/kg Li A. et al., 2013	30 mg/kg Wu et al., 2013
Alo	Cells	5 μM Hu et al., 2014	20 μM Hu et al., 2014
	Mice	Not reported	Not reported
	Rats	Not reported	Not reported
Rhe	Cells	10 μM Domagala et al., 2006	200 μM Fernand et al., 2011
	Mice	20 mg/kg Yu et al., 2015	80 mg/kg Yu et al., 2015
	Rats	100 mg/kg Ji et al., 2005	400 mg/kg Hou et al., 2012

## Conclusions

It is easy to find out not all the SHXXT constituents receive deep-enough investigation on their anti-inflammatory effect, the interaction between main SHXXT constituents and targets outside the nucleus get most focus. Besides, any drug elevating the absorption of Rhe, Ber, and Bae can be employed to promote oral bioavailability of SHXXT. Even though evidence shows P-gp, BCRP, and MRP really are inhibited while reports rarely cover the effect of SHXXT constituents on OCTs or SGLT. Hence, further investigation at these two levels is required to fully explain the mutual reinforcement relationship of RR, RC, and RS.

## Author contributions

JW: Prepare the manuscript; YH and LX: Search for the literatures; SL and YY: Draw the figures; XC and YZ: Do the summing work and accomplish the tables; WH: Polish language; XM and PW: Corresponding authors.

### Conflict of interest statement

The authors declare that the research was conducted in the absence of any commercial or financial relationships that could be construed as a potential conflict of interest.

## Abbreviations

ADM: adriamycin amycin
Akt: Protein kinase B; extracellular signal-regulated kinase
AMPK: 5′ AMP-activated protein kinase
Ang II: angiotensin II
AP-1: activator protein 1
ATF: Activating transcription factor
BCRP: breast cancer resistance protein
CaMK: Calcium/calmodulin- dependent kinase
CHOP: C/EBP homologous protein 10
CK: ginsenoside metabolite compound K
CMC-Na: caboxy methyl cellulose
CREB: cAMP response element-binding protein
Dex: Dexamethasone
Dox: Doxorubicin
DSS: dextran sulfate sodium
ERK: extracellular signal-regulated kinase
GSK3β: Glycogen synthase kinase 3 beta
HCM: hypercholesterolemic
HG: high glucose
HO-1: HMOX1heme oxygenase (decycling) 1
I/R: ischemia/reperfusion
IKK: IκBα kinase
IκBα: inhibitor of nuclear factor κBα
iNOS: inducible nitric oxide synthase
IRAK: Interleukin-1 receptor-associated kinase
IRF3: Interferon regulatory factor 3
IRS-1: Insulin receptor substrate 1
JAK: junas kinase
JNK: c-Jun NH2-terminal kinase
LKB1: liver kinase B1
LPS: lipopolysaccharide
MAPK: mitogen-activated protein kinase
MAPKAPK2: MAP kinase-activated protein kinase 2
MEK: Mitogen-activated protein kinase kinase
MRP: multidrug resistance associated protein
mTOR: mammalian target of rapamycin 2
MyD88: Myeloid differentiation primary response gene 88
NIK: NF-κB inducing kinase
Nrf2: Nuclear factor (erythroid-derived 2)-like 2
OCT: organic cation transporter
OGD: oxygen–glucose deprivation
PDTC: Pyrrolidine dithiocarbamate
P-gp: P-glycoprotein
PI3K: phosphoinositide 3-kinase
PMA: Phorbol-12-myristate-13-acetate
Poly(I:C): Polyinosinic:polycytidylic acid
PPAR-γ: peroxisome proliferator-activated receptor γ
Raf: RAF proto-oncogene serine/threonine-protein kinase
SAP: severe acute pancreatitis
SGLT1: Na+-dependent glucose transporter
SO: sham operation
SS: sodium salicylate
ST: sodium taurocholate
STAT: signal transducer and activator of transcription
TAK1: transforming growth factor-b-activated kinase
TLR-4: toll-like receptor
TNBS, 2, 4: 6-trinitrobenzene sulfonic acid
TNFR1: tumor necrosis factor receptor 1
TRADD: Tumor necrosis factor receptor type 1-associated death domain protein
TRAF: TNF receptor associated factors
TRM: epilepsy-like tremor
Tyk2: Non-receptor tyrosine-protein kinase 2
VSMC: vascular smooth muscle cell
WKY: Wistar-Kyoto
WT: wild type.
